# Relationship Between Cancer Related Fatigue, Physical Activity Related Health Competence, and Leisure Time Physical Activity in Cancer Patients and Survivors

**DOI:** 10.3389/fspor.2021.687365

**Published:** 2021-08-05

**Authors:** Maximilian Koeppel, Carlotta Körbi, Renate M. Winkels, Kathryn H. Schmitz, Joachim Wiskemann

**Affiliations:** ^1^Institute of Sport Science, Heidelberg University, Heidelberg, Germany; ^2^Public Health Sciences, Penn State College of Medicine, Hershey, PA, United States; ^3^Working Group Exercise Oncology, Division Medical Oncology, National Center for Tumordiseases Heidelberg and Heidelberg University Hospital, Heidelberg, Germany; ^4^Division of Human Nutrition and Health, Wageningen University, Wageningen, Netherlands

**Keywords:** cancer, cancer related fatigue, physical activity, physical activity related health competence, physical activity promotion among women in difficult life situations

## Abstract

**Background:** A large body of evidence supports the positive effects of leisure time physical activity (LTPA) and exercise on cancer survivors. However, only a fraction of survivors manages to attain international PA recommendations. This can be attributed to several external and internal barriers toward PA those patients seem to encounter, with cancer related fatigue (CRF) being the most reported internal barrier. Nevertheless, self-efficacy and knowledge about the utilization of LTPA can serve as facilitators of PA, which also correspond to certain constituents of physical activity related health competence (PAHCO). Since PAHCO is not investigated in cancer survivors we investigated if PAHCO can mediate the negative relationship between CRF and LTPA.

**Methods:** We surveyed 398 cancer survivors with different cancer types and therapy status. The patients completed the EORTC QLQ-FA12 (EORTC FA12) to assess CRF, the PAHCO questionnaire to assess PAHCO and the SQUASH to assess LTPA. We followed a two-step structural equation modeling approach. The first step established the measurement model, in the second step we fitted the mediation model. Since 163 patients chose not to answer the SQUASH, the mediation model was only fitted to the data of the remaining 235 participants.

**Results:** The proposed measurement model of the PAHCO offered an excellent fit. We found small to moderate positive associations between the PAHCO dimensions and the LTPA, and negative moderate relationships between the PAHCO and the EORTC FA12 dimensions. We did not observe a relationship between the EORTC FA12 dimensions and the LTPA (*p* > 0.05). The hypothesized mediation models did not display an appropriate fit.

**Conclusion:** The PAHCO confirmed its factorial validity; furthermore, it appears to have a positive relationship to LTPA. Therefore, integrating psycho-educational aspects can be beneficial in order to increase the PAHCO in exercise interventions. Because of the cross-sectional character of this study, causal conclusions are not suitable, therefore the longitudinal relationships of LTPA, CRF, and PAHCO require further investigation.

## Introduction

A large body of evidence supports the positive effects of physical activity (PA) and exercise for cancer patients all along the cancer trajectory (Fong et al., 2012; Furmaniak et al., 2016; Christensen et al., 2018). Exercise has shown to increase physical fitness (Strasser et al., 2013; Scott et al., 2018; Sweegers et al., 2019), health related quality of life (Mishra et al., 2012; Buffart et al., 2017) and improvement in cancer-related fatigue (CRF; Mustian et al., 2017; Van Vulpen et al., 2020). Prior to initial treatment, exercise can improve the fitness of cancer patients and reduce the likelihood of certain complications (Vermillion et al., 2018). During treatment, exercise has shown preventive health effects on cardio- and neurotoxicity (Chen et al., 2017; Kleckner et al., 2018); furthermore, it is closely associated with reducing cancer mortality and the probability of recurrence (Cormie et al., 2017; Friedenreich et al., 2020). Despite the benefits of regular PA, cancer patients tend to reduce their PA-levels after their diagnosis (Mason et al., 2013; Pugh et al., 2020) and only a minority of cancer survivors meet the PA guidelines (Blanchard et al., 2008; Lin et al., 2019). This can be contributed to particular internal as well as external barriers (e.g., the availability of programs and qualified facilities) cancer patients have to overcome, such as the frequently reported internal barrier of CRF (Brunet et al., 2013; Hardcastle et al., 2018).

CRF is one of the most common and distressing symptoms reported by cancer patients, produced by the cancer as well as its treatment (Bower, 2014; Berger et al., 2015). It is defined as a feeling intensive physical, emotional and cognitive exhaustion, without getting relieve after resting or sleeping (Weis, 2011). By the time cancer is diagnosed, 40% of patients already report signs of CRF (Rüffer, 2019). This proportion goes up to 60–100% during cancer treatments like chemotherapy and radiotherapy (Weis, 2011). CRF may even continue to impact the lives of long-term survivors for up to 10 years, as noted in 25–33% of cases (Bower, 2014). As of right now, there is no promising pharmacological treatment for CRF (Bower, 2014), with the most effective approach to mitigate CRF being PA (Mustian et al., 2017). This means cancer patients are often trapped in a vicious circle, since CRF prevents PA and thus increases CRF symptoms.

Patients need certain skills, knowledge and motivation (Schmid et al., 2020), i.e., they need competence, in order to positively engage in an active lifestyle. Thus, Pfeifer et al. (2013) developed the model of domain-specific physical activity-related health competence (PAHCO), inspired by the health model of Lenartz (2012), which focuses on promoting person-oriented competencies that enable a person to integrate an effective PA level into daily life. It contains three sub-competences (movement competence, control competence, and PA-specific self-regulation competence). Movement competence is a necessary condition that enables a person to realize the health benefits of exercise and everyday PA. Basically, movement competence comprises of motor skills and abilities. Control competence describes the degree a person is capable of utilizing their knowledge, based on the positive effects of exercise, how to implement health enhancing PA in their daily life, and how to control physical load in regards to their body signals, such as breathing, perceived exertion or heart rate. The third sub-competence, PA-specific self-regulation competence, encompasses the motivational and volitional abilities of a person to engage in health enhancing PA and implementing it into daily life (Sudeck and Pfeifer, 2016). Each of these sub-competencies contributes to regular health-promoting activity behavior. Sudeck and Pfeifer proposed a questionnaire to operationalize PAHCO, which predicts the PA-levels of patients undergoing medical rehabilitation (Sudeck and Pfeifer, 2016; Carl et al., 2020a).

A typical characteristic of CRF is its impairing effect on motivation (Brunet et al., 2013; Hardcastle et al., 2018); furthermore, CRF shows a positive relationship with depression (Bower et al., 2000; Brown et al., 2013). Considering that motivation and volition help using PA as constituents of the PAHCO model, PAHCO can play an important role in the correlation between CRF and LTPA. Since physical fatigue (PF) shows a certain sensitivity to PA (Van Vulpen et al., 2016), we would expect the relationships to be the strongest for this fatigue dimension. Because we were interested in the voluntary engagement of PA and exercise, in the hypotheses we focused on leisure time physical activity (LTPA). We therefore derived following five hypotheses.

- H_1_: The CRF dimensions display negative correlations with LTPA
- H_2_: The PAHCO dimensions display positive correlations with LTPA
- H_3_: The CRF dimensions display negative correlations with PAHCO dimensions
- H_4_: The PAHCO dimensions mediate the impact of CRF on LTPA
- H_5_: The relationships are strongest for PF.

We first investigated the factorial validity of the PAHCO questionnaire in an US based sample, since the PAHCO questionnaire has thus far only been investigated in German speaking populations (Sudeck and Pfeifer, 2016), afterwards we tested the hypotheses in a structural model.

## Materials and Methods

### Participants

Patients with any kind of cancer who were meeting the following inclusion criteria were eligible to participate in the study: ≥18 years, mobile enough to conduct exercise, and able to follow the study instructions. Patients were approached and asked to participate when they came to their treatment or follow-up.

### Procedure

The current study followed a cross-sectional design conducted at the Penn State Cancer Institute in Hershey PA, USA. The study protocol was approved by the Penn State College of Medicine Internal Review Board (Study ID: HRP-591-PEXO, Clinical Trials.gov: NCT04328038). All participants signed a written informed consent form before completing the survey. Patients were recruited in the waiting rooms of the hospital (Infusion Suite, Radiation Oncology, Surgery), where they received and completed the paper-and-pencil survey. In only a few cases the questionnaires were taken home and returned at the patient's next visit. The survey consisted of demographics and medical information, the PAHCO questionnaire (Sudeck and Pfeifer, 2016), the Emotion Thermometers (Mitchell et al., 2010), the Perception of Health Scale (Diamond et al., 2007), the BRIEF Health Literacy Screening Tool (Haun et al., 2009), the EORTC QLQ-C30 (Fayers et al., 2002), EORTC QLQ_FA12 (EORTC FA12) (Weis et al., 2017) and the short questionnaire to assess health-enhancing physical activity (SQUASH) (Wendel-Vos et al., 2003).

In this analysis, the PAHCO questionnaire, EORTC FA12 and SQUASH are reported for being of particular interest while the other questionnaires only served as material for missing data imputation. The time to complete the survey ranged between 15 and 20 min.

### Measures and Materials

#### The EORTC QLQ-FA12

The EORTC FA12 is a multidimensional self-reporting screening tool for assessing the extend of cancer-related fatigue. The tool was developed by the EORTC quality of life group and is used in conjunction with the EORTC QLQ-C30 (Weis et al., 2017). The questionnaire divides the CRF into three subscales using a total of 12 items: physical CRF (PF) (five items), emotional CRF (EF) (three items), and cognitive CRF (CF) (two items). The remaining two items serve as global indicators for impairment in performing daily life activities as well as the social sequelae of CRF, but they do not belong to a single subscale. All items were answered according to a four-stage Likert scale [from “not at all” (1) to “very much” (4)]. Reported Cronbach's alpha were good for all three dimensions with 0.88–0.90 for PF, 0.87–0.88 for EF and 0.79–0.82 for CF (Weis et al., 2017).

#### The Physical Activity Related Health Competence Questionnaire

The questionnaire is based on the PAHCO-Model outlined above and supposed to assess specific facets of the PAHCO, specifically addressing an individual's aptitude to effectively utilize physical activity to optimize their overall health. The questionnaire consists of 13 items comprised of three latent factors: PA-specific mood regulation (MR) (four items), control competence for physical training (CC) (six items) and PA-specific self-control (SC) (three items) (Sudeck and Pfeifer, 2016). In contrast to the PAHCO-Model, the PAHCO Questionnaire has no items to assess movement competence but instead focuses on the implementation (SC) and utilization (MR) of health enhancing PA, the control of physical load *via* body signals, as well as knowledge about the effects (CC). All items were answered on a four-stage Likert scale with possible responses ranging from “disagree completely” (1) to “agree completely” (4). Cronbach's alpha was good for all three dimensions with 0.89 for MR, 0.84 for CC and 0.78 for SC in patients undergoing exercise therapy and 0.88 for MR, 0.80 for CC and 0.80 for SC in people participating in health sports (Sudeck and Pfeifer, 2016). The original PAHCO questionnaire was developed and validated on two German samples (Sudeck and Pfeifer, 2016). However, the authors provided a version translated into English in their original publication. We used this translation and had it back translated into German by a member of the Penn State College of Medicine Internal Review Board who is both an English and German native speaker.

#### The Short Questionnaire to Assess Health-Enhancing Physical Activity

The SQUASH, a commonly used instrument to assess PA behavior in adults compares the physical activity levels of individuals and evaluates compliance to physical activity guidelines (Nicolaou et al., 2016). The SQUASH was developed by the Dutch National Institute of Public Health and the Environment (Wendel-Vos et al., 2003). It relies on self-reports, assessing 4 main domains: (a) commuting activities, (b) leisure time activities, (c) household activities, and (d) activities at work and school, which are evaluated based on an average week. The participants rate the amount of time they spent on each domain using three main queries: days per week, average time per day, and intensity (effort). To quantify the intensity of the activity, a metabolic equivalent task (MET) value, based on Ainsworth's compendium of PA (Ainsworth et al., 2000) is assigned to the activities, depending on the effort reported, the activities receive an intensity score and total score.

### Data Analysis

Descriptive statistics (i.e., means, standard deviations, inter quartile ranges, skewness, and kurtosis) were calculated. Linearity and collinearity between the single items were assessed by comparing bivariate Pearson-, Spearman-, and Kendall-tau-b correlation coefficients; in addition, optical bivariate scatter plots where we added to the Likert-scale items to increase interpretability (Gelman and Hill, 2007).

### Questionnaires

The analysis of the hypothesized mediation model involved a two-step process. In the first step a confirmatory factor analysis (CFA) was conducted to investigate the factorial validity of the proposed measurement models. In the second step the structural model was established (Anderson and Gerbing, 1988).

Items from both questionnaires show a good agreement regarding the three correlation coefficients and therefore indicate linearity in their relationships, which is supported by the scatter plots. The normality assumption was tested *via* Shapiro–Wilk-Test for univariate normality and Mardia-Test for multidimensional normality. Univariate as well as multivariate normality was violated for all items in both questionnaires (*p* < 0.001).

#### Leisure Time Physical Activity

The univariate normality of the LTPA data was tested with the Shapiro–Wilk Test. For the outlier identification we conducted a qualitative approach by visually examining the LTPA distribution. Three values beyond 150 MET-h/week were identified which did not fit into the remaining distribution and thus were omitted from the data set. The remaining LTPA-data violated the assumption of normality (*p* < 0.001). Because of the violation of the normality assumption, we conducted a Box-Cox transformation by the power of 1/6 with the LTPA-data, resulting in an improved approximation of the normal distribution (*p* = 0.055). The raw data and the transformed data showed a Pearson-correlation of *r* = 0.89.

#### Missing Data

We excluded cases when the entire questionnaire was missing and acknowledged one missing entry for age and eight missing entries for gender. In the remaining data set 2–7 (0.5–1.7%) answers for the PAHCO items were missing and for the EORTC-FA 12 1–2 (0.25–0.5%) answers were missing. The data showed a general missing data pattern. We investigated the appropriateness of the variables for multiple imputations *via* influx and outflux diagrams (Van Buuren, 2018). All variables except of LTPA were appropriate for the imputation procedure. The comparison of the original and imputed variables *via* Mann–Whitney-*U*-Test found no evidence for differences between the original and the imputed data sets (Range of *p* = [0.79, 1.0]).

#### Measurement Model Estimation and Model Fit

The measurement models were estimated with the variance of the latent factors set to 1.0, this allowed us to compare the observed factor loadings to the ones reported in the original publications. Because of the violation of the normality assumption, we applied the maximum likelihood approach with a 10,000-replication bootstrap with 95% bias corrected confidence interval as well as the DWLS estimation, treating the items of the questionnaires as ordinal. Evaluation of the model fit was based on chi-squared (χ^2^) tests, the chi-square-degrees of freedom ratio (χ^2^/df), the Root Means Square Error of Approximation (RMSEA), the standardized Root Mean Square Residual (SRMR) as well as the Comparative Fit Index (CFI) and the Tucker Lewis Index (TLI). For the χ^2^/df a value <3 was considered as acceptable (Iacobucci, 2010). For CFI and TLI values >0.90 were considered acceptable and >0.95 considered as good, for RMSEA and SRMR values <0.06 were considered as good and <0.08 as acceptable (Hu and Bentler, 1999). The measurement model which displayed the better fit was included into the structural model. Scale reliability was measured *via* McDonald's omega as an alternative to Cronbach's alpha, appreciating variability in factor loadings (Dunn et al., 2014). Discriminant validity between the factors was assessed using the Fornell–Larcker-Criterion. In this case the average variance extracted (AVE) should be larger than the highest squared inter-factor correlation corresponding to the variance shared by these two factors, i.e., the variance of the latent variable which can be attributed to its the indicators instead of measurement error (Fornell and Larcker, 1981; Rönkkö and Cho, 2020).

#### Structural Model

**Figure 1:**
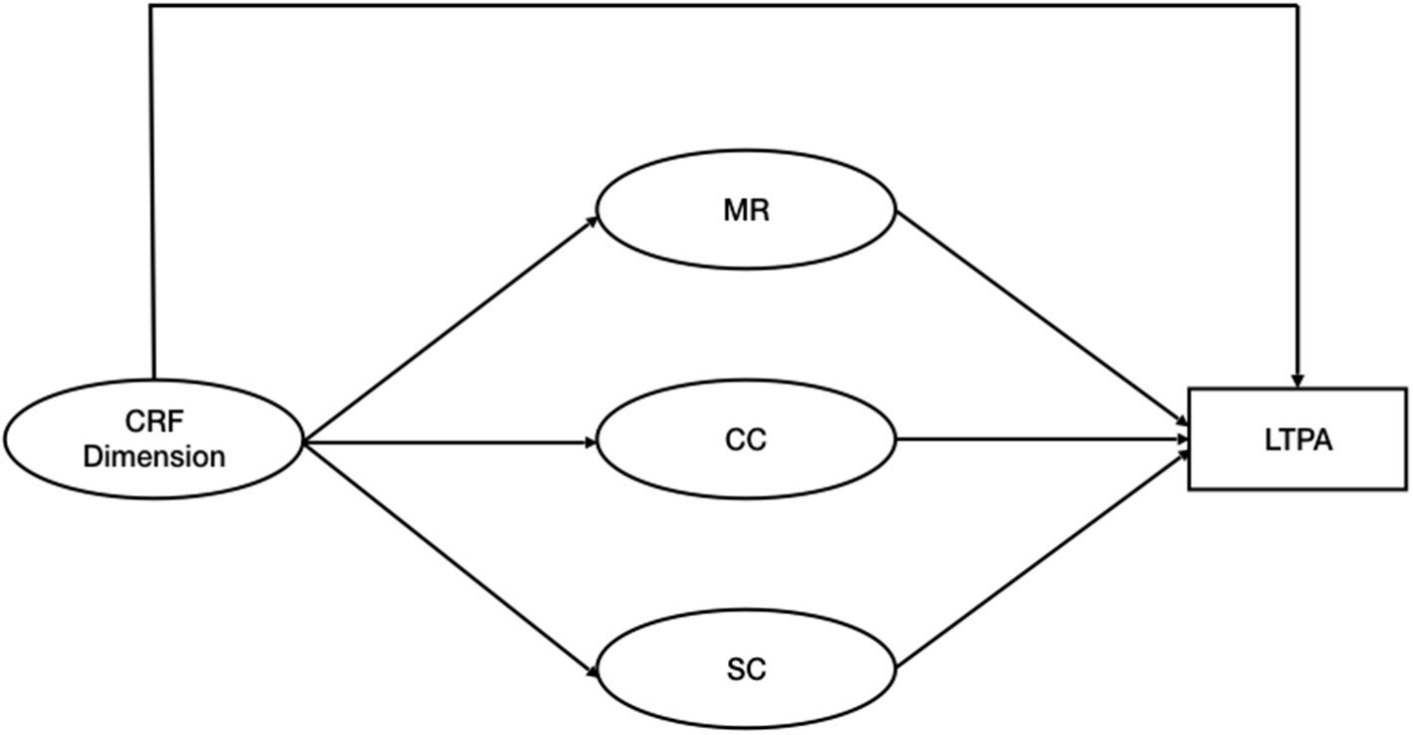
Hypothesized mediation model. CRF, Cancer Related Fatigue; MR, Physical Activity Specific Mood Regulation; CC, Control Competence for Physical Training; SC, Physical Activity Specific Self Control; LTPA, Leisure Time Physical Activity.

The three PAHCO dimensions as well as the three Fatigue dimensions were considered latent variables, as postulated in the literature (Sudeck and Pfeifer, 2016; Weis et al., 2017). The transformed LTPA was considered a manifest variable. To test the hypotheses we fitted three separate models for each CRF dimension where the relationship between CRF and LTPA is mediated by the three PAHCO (Figure 1).

We calculated direct, indirect and total effects. To evaluate the model fit the same parameters and criteria as outlined for the measurement models were applied.

All analysis were conducted in R (Team, 2013). Multiple Imputation was done *via* the R-package mice (Van Buuren and Groothuis-Oudshoorn, 2011), CFA and structural model were conducted *via* R-package lavaan (Rosseel, 2012).

## Results

### Participants

**Figure 2:**
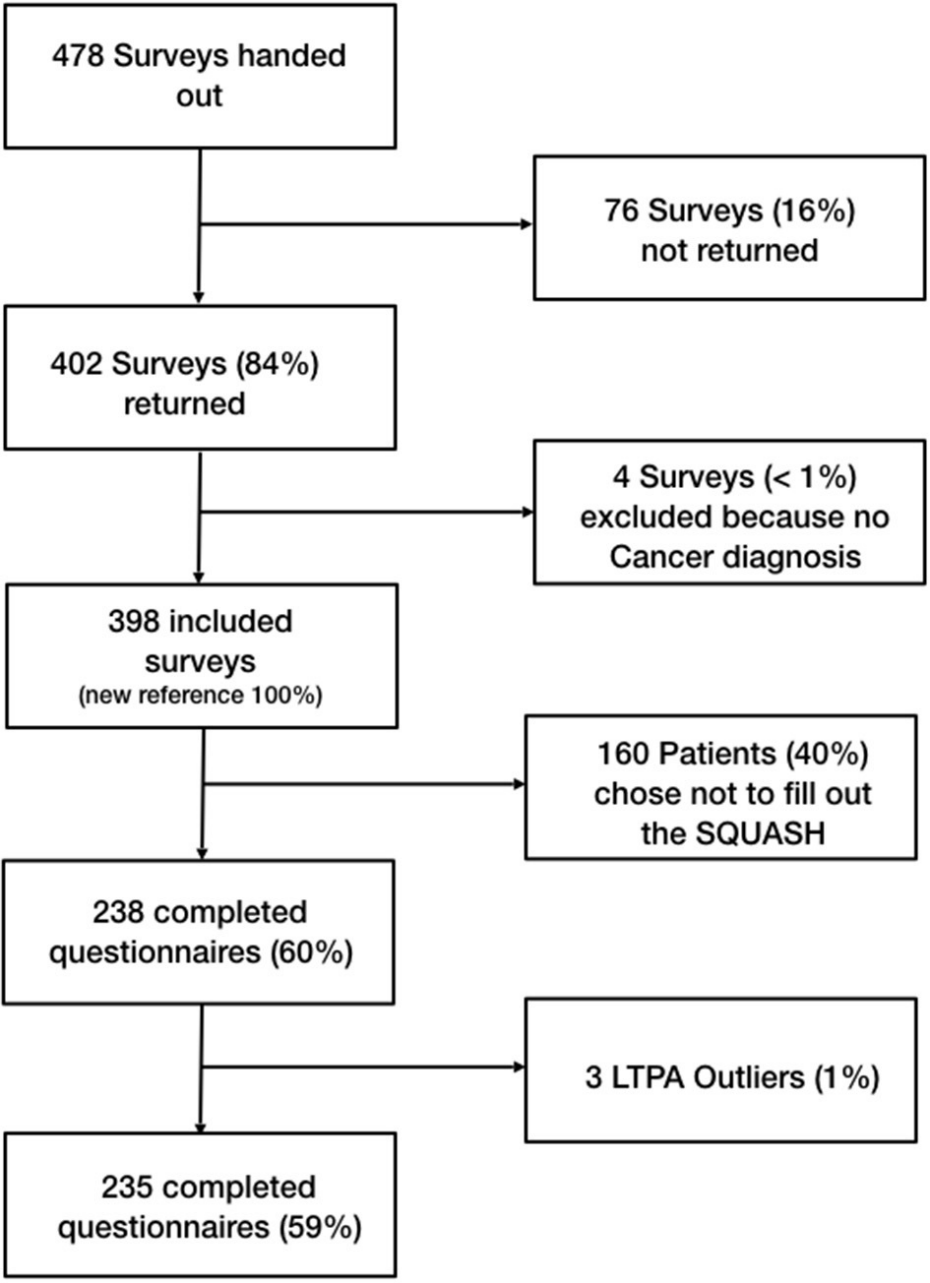
Patient flow.

In total a sample of 398 cancer patients (169 men and 221 women, 8 unknown) were surveyed. Initially 478 surveys were handed out, resulting in a return rate of 84%. The mean age of the participants was 63 years, with the age ranging from 22 to 89 years. The sample spanned over all levels of educational achievement. Approximately half of all participants reported treatment with chemotherapy, while half of the participants reported undergoing radiotherapy. Forty-two percent of participants have already finished their tumor therapy, of which 28% finished their therapy more than 2 years ago. Twenty-two percent of participants were still undergoing therapy at the time the survey was completed. Twenty-six percent did not provide information about their treatment. Twenty-nine percent of the sample were breast cancer survivors, and 14% of participants did not provide information about their cancer site. Two participants were excluded for having no history of cancer (Table 1; Figure 2). One hundred and sixty-three participants chose not to answer the SQUASH questionnaire. These patients displayed significant differences (*p* < 0.05) in four of the 13 PAHCO items (Item 1–3, loading on MR and Item 11 loading on SC) as well as five items (Item 1–3 loading on PF and Item 9–10 loading on CF) of the EORTC FA12. In the case of the PAHCO items, the proportion who completed the SQUASH showed higher scores in each of the four items, indicating a higher PAHCO. Simultaneously, those ones who did not complete the SQUASH showed higher scores in each of the five concerned EORTC FA12 items, indicating higher levels of CRF.

**Table 1:** Patient characteristics.

**Characteristics**	***n***	**%**
Total	*398*	*100*
**Demographics**		
Age, mean ± SD, years	63.3 (± 12.5)	
***Sex***		
Female	221	55.5
Male	169	42.5
N/A	8	2
***Marital status***		
Married or living with a partner	265	66.6
Single	86	21.6
Widowed	37	9.3
N/A	10	2.5
***Race***		
White	364	91.5
Black or African American	13	3.2
Other	5	1.3
N/A	16	4
***Education***		
HS graduate or less	128	32.2
Some college	128	32.2
College graduate or more	133	33.4
N/A	9	2.2
***Employment status***		
Retired/Not working	193	48.7
Still working	194	48.5
N/A	11	2.8
**Medical profile**		
***Primary cancer diagnosis***		
Breast	116	29.1
Melanoma	49	12.3
Colon	38	9.5
Prostate	34	8.5
Other	105	26.5
N/A	56	14.1
***Therapy***		
Chemotherapy	190	47.7
Radiotherapy	195	49
Immunotherapy	82	20.6
Hormonetherapy	62	15.6
Surgery	277	69.6
Therapy completed	165	41.5
Therapy completed 2 years ago	46	11.6
***Comorbidities***		
≥1 cardiovascular/pulmonic disorder	156	39.2
≥1 orthopedic/rheumatologic disorder	129	34.4
Diabetes	62	15.6
**Questionnaires**		
***EORTC FA12***	26.7 (20, 46.7)	
PF, Median (IQR)		
EF, Median (IQR)	11.1 (0, 33.3)	
CF, Median (IQR)	0 (0, 33.3)	
***PAHCO***		
MR, Median (IQR)	75.0 (58.3, 100)	
CC, Median (IQR)	66.7 (44.4, 83.3)	
SC, Median (IQR)	44.4 (33.3, 66.7)	
Physical activity	*n* = 235	59%
LTPA, Median (IQR)	11.2 (0, 36.9)	

*PF, Physical Cancer Related Fatigue; EF, Emotional Cancer Related Fatigue; CF, Cognitive Cancer Related Fatigue; PAHCO, Physical Activity Related Health Competence; MR, Physical Activity Specific Mood Regulation; CC, Control Competence for Physical Training; SC, Physical Activity Specific Self Control; LTPA, Leisure Time Physical Activity*.

### Measurement Models

The confirmatory factor analysis measured three latent factors for the PAHCO (MR, CC, and SC) as well as the EORTC FA12 (PF, EF, and CF). All three PAHCO and EORTC factors showed good reliability with McDonald's omega of 0.94 for MR, 0.93 for CC and 0.92 for SC and 0.94 for PF, 0.89 for EF and 0.84 for CF. In order to assess discriminant validity we looked at the AVE from the indicators of 0.85 for MR, 0.67 for CC, and 0.87 for SC for the PAHCO as well as 0.79 for PF, 0.77 for EF, and 0.84 for CF for the EORTC FA12. In comparison the squared factor correlations between the latent variables is 0.23 for MR and SC, 0.33 for MR and CC, 0.55 for CC and SC, 0.56 for PF and EF, 0.41 for PF and CF and 0.60 for EF and CF in case of the EORTC FA12. Thus, the tallest shared variance between the factors appears to be smaller than the smallest AVE. Of the four different measurement models, the DWLS estimation treating the EORTC FA12 items as ordinal and the PAHCO items as continuous (Model 2) showed the best fit [$\chi_{\left(275\right)}^{2}$ = 196, *p* = 1.00], with all fit criteria indicating good to excellent fit (Table 2). The second best fit is displayed by the model treating the PAHCO items as ordinal and the EORTC FA12 items as continuous (Model 3) [$\chi_{\left(275\right)}^{2}$ = 290, *p* = 0.251] The model treating all items ordinal (Model 4)showed a slightly worse fit than Model 2 and 3. The least fitting model was Model 1 that treated all items continuous [$\chi_{\left(275\right)}^{2}$ = 536, *p* = 0.251]. Thus, for all following analyses we opted for Model 2.

**Table 2:** Fit of the measurement models.

	**χ^2^**	**df**	***p*** **-value**	**χ^2^/df**	**CFI**	**TLI**	**RMSEA**	**RMSEA CI**	**SRMR**
Model 1	536	*275*	<0.001	1.95	0.928	0.915	0.064	0.056–0.072	0.050
Model 2	*290*	*275*	*0.251*	*1.05*	*0.999*	*0.999*	*0.016*	*<0.001– <0.001*	*0.051*
Model 3	*196*	*275*	*1.00*	*0.71*	*1.00*	*1.006*	*<0.001*	*<0.001–0.031*	*0.052*
Model 4	*347*	*275*	*0.002*	*1.26*	*0.998*	*0.997*	*0.034*	*0.021–0.044*	*0.057*

*χ^2^, chi-squared; df, degrees of freedom; χ^2^/df, chi-square-degrees of freedom ratio; CFI, Comparative Fit Index; TLI, Tucker Lewis Index; RMSEA, Root Means Square Error of Approximation; SRMR, Standardized Root Mean Square Residual*.

Latent factors within the same questionnaire showed moderate to strong positive associations with each other [0.5–0.7].

### Structural Models

**Figure 3:**
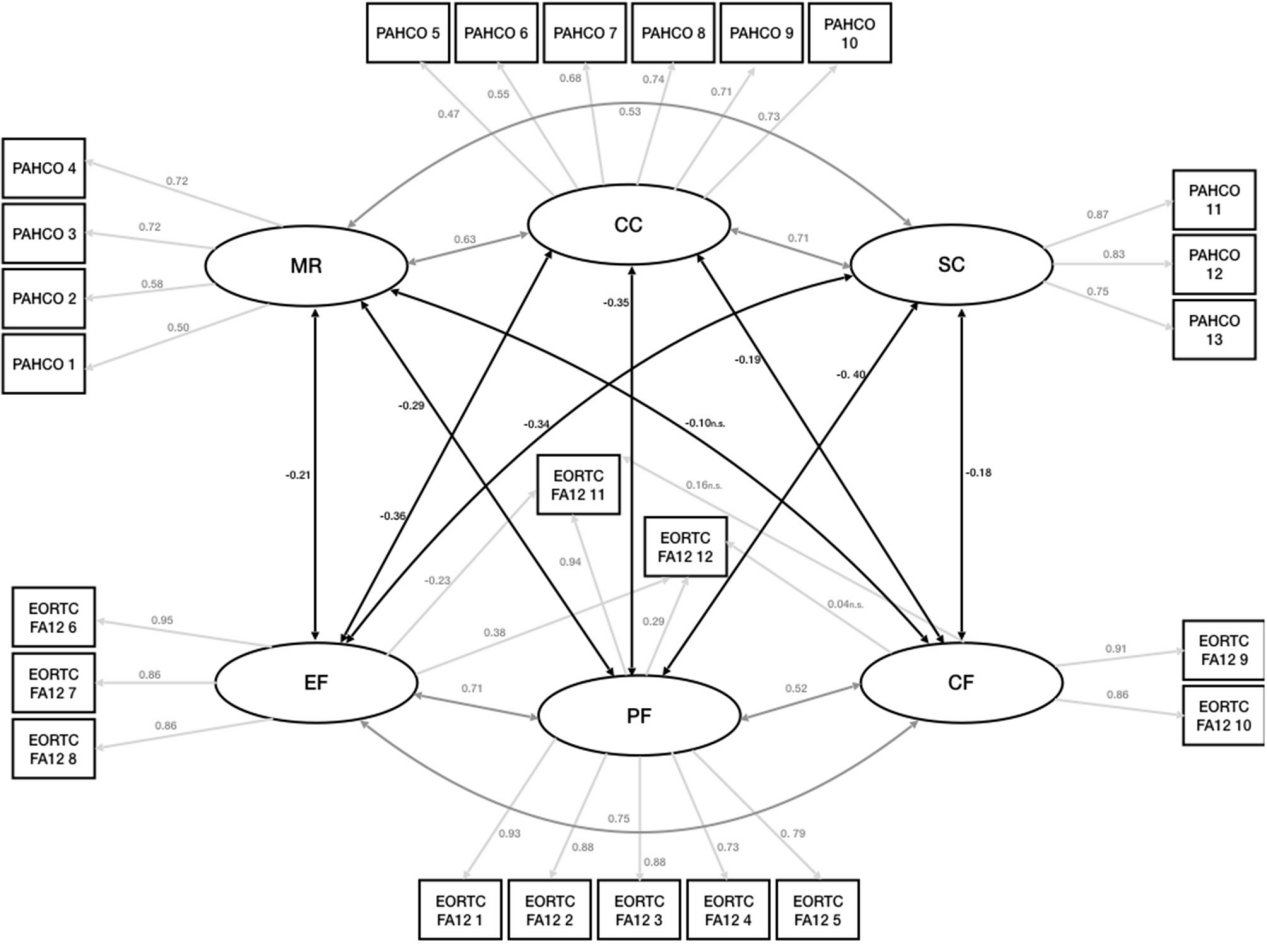
Measurement model and structural components. EORTC FA12, EORTC QLQ-FA12; PF, Physical Cancer Related Fatigue; EF, Emotional Cancer Related Fatigue; CF, Cognitive Cancer Related Fatigue; PAHCO, Physical Activity Related Health Competence; MR, Physical Activity Specific Mood Regulation; CC, Control Competence for Physical Training; SC, Physical Activity Specific Self Control.

No association between LTPA and the CRF dimensions was observed (*p* > 0.05) with respect to the borderline significant negative association of −0.15 (*p* = 0.063) between CF and LTPA (Rejection of H_1_). The latent factors showed small to moderate negative correlations between the questionnaires [0.2–0.4], i.e., the CRF dimensions were negatively associated with the PAHCO dimensions (Confirmation of H_3_) (Figure 3). Small to moderate positive correlations [0.18–0.27] for all PAHCO dimensions and LTPA were observed (Table 3) (Confirmation of H_2_).

**Table 3:** Correlation matrix of the latent factors and LTPA.

	**PF**	**EF**	**CF**	**MR**	**CC**	**SC**	**LTPA**
**PF**	1	0.707^**^	0.524^**^	−0.288^**^	−0.350^**^	−0.395^**^	−0.026
**EF**		1	0.746^**^	−0.205^*^	−0.358^**^	−0.336^**^	−0.057
CF			1	−0.095	−0.193^*^	−0.183^*^	−0.150^†^
MR				1	0.626^**^	0.530^**^	0.268^**^
CC					1	0.706^**^	0.188^*^
SC						1	0.181^*^
LTPA							1

** *p < 0.001*,

* *p < 0.05*,

† *p < 0.10; PF, Physical Cancer Related Fatigue; EF, Emotional Cancer Related Fatigue; CF, Cognitive Cancer Related Fatigue; PAHCO, Physical Activity Related Health Competence; MR, Physical Activity Specific Mood Regulation; CC, Control Competence for Physical Training; SC, Physical Activity Specific Self Control; LTPA, Leisure Time Physical Activity*.

The hypothesized models displayed an insufficient fit of the data regarding the mediation in all three CRF dimensions (PF, EF, and CF). Although the CFI and TLI showed acceptable to good fits, RMSEA, SRMR, chi^2^, and the chi/df-ratio did not meet any of the thresholds, therefore, the mediation models did not seem justified (Rejection of H_4_ and H_5_) (Table 4).

**Table 4:** Fit of the structural models.

	**χ^2^**	**df**	**χ^2^/df**	**CFI**	**TLI**	**RMSEA**	**RMSEA CI**	**SRMR**
PF	1,490	183	8.14	0.954	0.947	0.177	0.142–0.169	0.159
EF	957	146	6.55	0.964	0.958	0.156	0.147–0.166	0.142
CF	600	129	4.65	0.977	0.973	0.127	0.137–0.116	0.104

*χ^2^, chi-squared; df, degrees of freedom; χ^2^/df, chi-square-degrees of freedom ratio; CFI, Comparative Fit Index; TLI, Tucker Lewis Index; RMSEA, Root Means Square Error of Approximation; SRMR, Standardized Root Mean Square Residual; PF, Physical Cancer Related Fatigue; EF, Emotional Cancer Related Fatigue; CF, Cognitive Cancer Related Fatigue*.

## Discussion

This analysis was able to confirm the factorial validity for the American-English version of the PAHCO, involving US-American cancer patients and survivors. The loadings and correlations between the latent PAHCO variables in our model roughly coincides with the correlational structure of the German original publication, reporting slightly lower loadings on MR and CC as well as slightly higher loadings on SC. Furthermore, we identified a moderate positive association between PAHCO-dimensions and LTPA, which conforms to observations made in other populations and also confirms H_2_ (Sudeck and Pfeifer, 2016; Carl et al., 2020a). Of all three PAHCO dimensions, MR and LTPA showed the strongest association, while the association between LTPA and the other two remaining PAHCO dimensions had slightly smaller correlation coefficients. Ostensibly, the strong correlation between MR and LTPA can be attributed to the items loading on MR, addressing depression, stress and inner tension, which are elevated for cancer patients in comparison to the general public or even patients with other chronic diseases (Gil et al., 2012; Hartung et al., 2017; Rao et al., 2019). This may suggest that physical activity has an instrumental functionality when it comes to cancer patients since it helps them cope with psychological symptoms. It also goes in line with the recently published ACSM Roundtable on Physical Activity Guidelines for Cancer Patients (Campbell et al., 2019) which confirms a positive effect of exercise on depressive symptoms and anxiety. Nevertheless, the three correlation coefficients between LTPA and the PAHCO dimensions do not differ significantly from each other, considering their 95% confidence intervals, hence, conclusions must be drawn with caution and can be premature. According to H_3_, all PAHCO-dimensions displayed a negative relationship to the CRF-dimensions, however, MR showed the weakest correlation to either of the CRF dimensions, which are usually linked to mood disturbances and have a strong relation with depression (Bower et al., 2000; Brown et al., 2013).

A relationship between the CRF dimensions and LTPA has not been observed, which opposes the literature and H_1_, that exhibits a small to moderate negative associations between CRF and PA levels (Kummer et al., 2013; Galiano-Castillo et al., 2014; Romero et al., 2018). This could be partially explained by a possible selection bias due to a lower response rate to the SQUASH compared to the other questionnaires (about 59%). Pertaining the questionnaires, participants who chose not to answer the SQUASH scored significantly lower in three of the four items loading on MR, significantly higher in three of seven items loading on PF, and significantly higher on two of four items loading on CF. Thus, the patients who completed the SQUASH and were investigated for the final analysis presented a higher PAHCO and lower CRF than the others. Bearing in mind that the SQUASH items are more arduous to answer than the Likert-scaled questions of the other questionnaires, it appeared that people with a positive attitude toward PA in general and LTPA in particular would invest more effort into completing the survey.

We could not observe a ceiling effect or lack of variance in the LTPA-Data; though, a plausible correlation between the PAHCO-dimensions and PA as well as between the PAHCO dimensions and EORTC FA12 were established. Accordingly, we should have been able to identify a relevant association, despite the likelihood of selection bias.

A conceivable mediation for the effect of CRF on LTPA *via* PAHCO renders obsolete (H_4_ and H_5_) given the lack of association between the two variables. Considering the cross-sectional character of the study, derived conclusions should not indicate causation, and further investigations need to examine relationship between CRF, LTPA and the role of PAHCO in a longitudinal manner. Subsequently, the time-lagged cross-correlations between CRF and LTPA would reveal further information about the relationship between these variables along the cancer trajectory. Due to the decline in PA-levels patients experience following cancer diagnosis and treatment, finding the causes as well as potential interventions are of great interests. Besides its explanatory value, increasing PAHCO in patients could potentially increase adherence to given exercise programs and an enhanced active lifestyle. One of the most common reported intrinsic barriers of cancer patients toward LTPA is the lack of self-efficacy (Brunet et al., 2013; Phillips and McAuley, 2013; Ungar et al., 2016; Hardcastle et al., 2018). In a systematic review the perception of control over ones well-being was one of the most common facilitators of exercise (Clifford et al., 2018). Another review has shown a strong prediction of instrumental attitude and planning on LTPA levels (Vallance et al., 2008). In fact, all these constructs can be considered constituents of PAHCO.

For patient care, PAHCO could be used to assess the level of support patients require to initiate an exercise routine and LTPA. By stratifying patients in accordance with their PAHCO levels, exercise therapists would be able to provide educational instructions tailored particularly to every patient's needs. By increasing PAHCO, patients would presumably feel empowered to engage in exercise and LTPA independently. In regards to health literacy, cancer patients with lower levels of knowledge seemed to necessitate expert support more than patients with higher levels, who were also more likely to perform research themselves (Morris et al., 2013). Additionally, the stratification process may prevent the misallocation of resources by providing especially patients in need with the highest level of guidance. Consequently, PAHCO is a promising concept to promote PA in cancer patients, which would result in vital lifestyle changes and increased participation in exercise programs.

### Limitations

This study seems to have a couple of limitations. First, the cross-sectional character of the study does not allow for causal conclusions. Therefore, the results from a mediation approach, which has an underlying causal preposition, will have to be interpreted with caution. An opposing relationship seems possible as well, meaning the therapeutic effect of LTPA on CRF can be utilized more efficiently by people with higher levels of PAHCO. Further research is necessary in order to investigate the relationship between LTPA, PAHCO and CRF with an experimental approach. Furthermore, movement competence, one of the three major sub-competences of the PAHCO model, was not assessed by the PAHCO questionnaire. Accordingly, the PAHCO has not been assessed in its entirety. More recent PAHCO instruments have already made suggestions to measure this dimension (Carl et al., 2020b) and should be applied to further research. Additionally, we encountered an immense loss of information due to incomplete PA-questionnaires, hence, less laborious but still reliable instruments, that assess physical activity in cancer patients, have been implemented without increasing study logistics and costs considerably, as it would be the case for accelerometers.

## Data Availability Statement

The raw data supporting the conclusions of this article will be made available by the authors, without undue reservation.

## Ethics Statement

The studies involving human participants were reviewed and approved by PennState College of Medicine internal review board (Study ID: HRP-591-PEXO, Clincial Trials.gov: NCT04328038). The patients/participants provided their written informed consent to participate in this study.

## Author Contributions

MK: data collection, conducting the statistical analysis, and writing the manuscript. CK: data entry, descriptive statistics, and writing the manuscript. RW and KS: study design, coordination of data collection, and critical review of the manuscript. JW: study design, conducting the statistical analysis, and critical review of the manuscript. All authors contributed to the article and approved the submitted version.

## Conflict of Interest

The authors declare that the research was conducted in the absence of any commercial or financial relationships that could be construed as a potential conflict of interest. The reviewer SS declared a shared affiliation, with no collaboration, with the authors (CK and JW) to the handling editor at the time of review.

## Publisher's Note

All claims expressed in this article are solely those of the authors and do not necessarily represent those of their affiliated organizations, or those of the publisher, the editors and the reviewers. Any product that may be evaluated in this article, or claim that may be made by its manufacturer, is not guaranteed or endorsed by the publisher.

## Abbreviations

AVE: Average Variance Extracted
CRF: Cancer Related Fatigue
χ^2^: chi-squared
χ^2^/df: chi-square-degrees of freedom ratio
CF: Cognitive Cancer Related Fatigue
CFI: Comparative Fit Index
CFA: Confirmatory Factor Analysis
CC: Control Competence for Physical Training
df: degrees of freedom
EF: Emotional Cancer Related Fatigue
LTPA: Leisure Time Physical Activity
MET: Metabolic Equivalent Task
MR: Physical Activity Specific Mood Regulation
SC: Physical Activity Specific Self Control
PA: Physical Activity
PAHCO: Physical Activity Related Health Competence
PF: Physical Cancer Related Fatigue
RMSEA: Root Means Square Error of Approximation
SRMR: Standardized Root Mean Square Residual
TLI: Tucker Lewis Index.
